# Oral Health and Oral Health-Related Quality of Life in Patients with Primary Sjögren’s Syndrome

**DOI:** 10.3390/medicina59030473

**Published:** 2023-02-27

**Authors:** Sanja Vujovic, Jana Desnica, Momir Stevanovic, Sara Mijailovic, Radisa Vojinovic, Dragica Selakovic, Nemanja Jovicic, Gvozden Rosic, Dragan Milovanovic

**Affiliations:** 1 Department of Dentistry, Faculty of Medical Sciences, University of Kragujevac, Svetozara Markovića 69, 34000 Kragujevac, Serbia; 2 Department of Medical Statistics and Informatics, Faculty of Medical Sciences, University of Kragujevac, Svetozara Markovića 69, 34000 Kragujevac, Serbia; 3 Department of Radiology, Faculty of Medical Sciences, University of Kragujevac, Svetozara Markovića 69, 34000 Kragujevac, Serbia; 4 Department of Physiology, Faculty of Medical Sciences, University of Kragujevac, Svetozara Markovića 69, 34000 Kragujevac, Serbia; 5 Department of Histology and Embryology, Faculty of Medical Sciences, University of Kragujevac, Svetozara Markovića 69, 34000 Kragujevac, Serbia; 6 Department of Pharmacology and Toxicology, Faculty of Medical Sciences, University of Kragujevac, Svetozara Markovića 69, 34000 Kragujevac, Serbia

**Keywords:** oral health, oral health-related quality of life, oral manifestations, primary Sjögren’s syndrome, xerostomia

## Abstract

*Background and Objectives*: Oral disorders, frequently observed in patients with primary Sjögren’s syndrome, can profoundly affect patients’ daily lives and well-being, as oral health represents a fundamental part of general health. Saliva plays an essential part in maintaining and protecting oral health, so the decrease in its quantity and quality leads to chronic oral discomfort alongside a broad spectrum of problems. The objective of the present study was to evaluate the oral health of patients with primary Sjögren’s syndrome and establish its effect on the different domains of their oral health-related quality of life (OHRQoL). *Materials and Methods*: The research was designed as an observational case–control study with prospective data collection. Eighty patients, divided into two groups based on their oral status, participated in the study. All subjects underwent a complete oral examination. The OHRQoL was assessed using the Oral Health Impact Profile-14 (OHIP-14). *Results*: The most prevalent oral manifestation was exfoliative cheilitis, while 30% of subjects complained of chewing and swallowing difficulties. The OHIP-14 summary score was significantly higher in the patients with oral lesions (26.0 (5.0) vs. 17.0 (4.0), respectively; *p* < 0.001). Oral manifestations, systemic involvement, medication, and periodontal indexes were significantly associated with OHIP-14 scores. *Conclusions*: Patients with oral alterations had a substantially decreased OHRQoL. These findings emphasize the importance of oral diseases for patients’ well-being. Therefore, it is essential for dentists to be included in the multidisciplinary teams managing primary Sjögren’s syndrome, as improving patients’ oral status would lead to better oral health and enhanced OHRQoL.

## 1. Introduction

Primary Sjögren syndrome (pSS) is a chronic autoimmune rheumatic disease characterized by progressive focal lymphocytic infiltration of the exocrine glands, with an estimated prevalence of 0.05–1% in the European population [1]. In particular, salivary and lacrimal glands are the most affected, making xerostomia (dry mouth) and xerophthalmia (dry eyes) the main hallmarks of the pSS [2,3]. Up to 50% of patients might develop systemic complications such as arthritis, vasculitis, peripheral neuropathy, interstitial lung disease, and interstitial nephritis [4]. Severe fatigue, muscle/joint pain, and psychological distress are also often reported [5].

Saliva plays an essential part in maintaining and protecting oral health, so the decrease in its quantity and quality leads to chronic oral discomfort alongside a broad spectrum of problems [6]. Some of the most common oral manifestations associated with pSS are dental caries, atrophy of the oral mucosa, glossitis, oral ulcers, and fungal infections [7,8,9]. Due to hyposalivation, patients frequently experience chewing, swallowing, and speaking difficulties [8]. Additional issues include chemosensory dysfunction, halitosis, and trouble wearing dentures [8,9].

Oral disorders can profoundly affect patients’ daily lives and well-being, as oral health represents a fundamental part of general health. Therefore, serious efforts have been made in the past two decades to develop specific instruments for the evaluation of oral diseases’ impact on various aspects of the oral health-related quality of life (OHRQoL) [5]. Previous studies have demonstrated that xerostomia results in the deterioration of OHRQoL, so it can be hypothesized that patients suffering from pSS will also report physical, psychological, and social consequences due to oral problems [10,11].

The aim of this article was to evaluate the oral health of pSS patients and establish its effect on the different domains of their OHRQoL.

## 2. Materials and Methods

### 2.1. Study Design and Participants Selection

The research was designed as an observational case–control study with prospective data collection and performed at the Rheumatology Clinic of the University Clinical Centre of Kragujevac and the Dentistry Department of the Faculty of Medical Sciences, University of Kragujevac. The study protocol was approved by the Ethics Committee of the University Clinical Centre of Kragujevac (decision number 01/20-657), and it was conducted in accordance with the Helsinki Declaration of 1964 and its later amendments. The period of recruitment was between July 2021 and September 2022. The study included 80 patients (age over 18) with the diagnosis of pSS, according to the American College of Rheumatology/European League Against Rheumatism (ACR-EULAR) classification criteria [12]. These criteria include ocular symptoms (minimum one: dryness of the eyes persisting for more than 3 months, recurrent sandy-gritty eye irritation, or the need to use artificial tears for more than 3 months), oral symptoms (minimum one: dryness of the mouth persisting for more than 3 months, recurrent swelling of the salivary glands, or the need to sip fluid with swallowing), ocular signs (positive Schirmer’s test or Rose–Bengal score of ≥4), oral signs (positive result in one of the following tests: salivary scintigraphy, parotid sialography, or unstimulated salivary flow), focus score ≥ 1 in a minor salivary gland biopsy, and positive autoantibodies against SS-A and/or SS-B [13]. Patients were diagnosed with pSS if 4 out of 6 criteria were fulfilled; positive salivary gland biopsy or the presence of anti-Ro/SSA and anti-La/SSB autoantibodies are required for the diagnosis. The subjects under the age of 18, patients with systemic connective tissue diseases, mental disorders, malignant neoplasms, pregnant women, and active smokers were excluded from the study. Patients who met the inclusion criteria were invited to participate in the research. All subjects signed the written informed consent before enrollment. Participants were assigned into two groups based on their oral status. One group (case) consisted of subjects with oral lesions and symptoms, while the other group (control) comprised patients who did not experience any oral manifestations and symptoms besides the subjective feeling of dryness. Participants’ socio-demographic data and pSS clinical characteristics were recorded with a specially designed questionnaire for research purposes.

### 2.2. Sample Size

The sample size was calculated using G*Power software (v3.1.9.7; Faculty of Mathematics and Natural Sciences, Dusseldorf, Germany) based on the data on mean values of OHIP-14 in pSS patients from a recent study by Fernández Castro et al. [14]. In order to detect a difference in proportions of at least 30% between the two groups at a statistical significance level of 0.05 and power of 80%, a sample size of 40 subjects in each group was needed to meet the study requirements.

### 2.3. Oral Examination

The complete oral examination took place at the Dentistry Department of the Faculty of Medical Sciences. All patients were examined by the same doctor of dental medicine due to the objectivity and consistency of the gathered data. The investigator used a graduated periodontal probe and a standard dental mirror for each examination. Diagnosis of the oral manifestations was established according to WHO guidelines [6]. Lesion type, localization, clinical appearance, signs, and symptoms were documented. Additionally, the following periodontal parameters were collected and recorded: Community Periodontal Index of Treatment Need (CPITN), plaque index (PI), gingival index (GI), and sulcus bleeding index (SBI).

### 2.4. Assessment of the Oral Health-Related Quality of Life

OHRQoL was evaluated using the short Serbian version of the Oral Health Impact Profile (OHIP-14) questionnaire [15]. OHIP-14 represents one of the most widely applied tools worldwide for OHRQoL assessment in routine practice and clinical research [7]. It is a self-administered instrument that consists of 14 items divided into 7 domains: functional limitation, physical pain, psychological discomfort, physical disability, psychological disability, social disability, and handicap [7]. Questions are scored on a 5-point Likert scale, with response options spanning from 0 to 4 (0—never, 1—hardly ever, 2—occasionally, 3—fairly often, and 4—very often). The single summary score may range between 0 and 56, with higher results implicating poorer OHRQoL [7].

### 2.5. Statistical Analysis

Statistical analysis was performed in the SPSS statistical program, version 22 for Windows (IBM SPSS Statistics 22, Armonk, NY, USA). A descriptive method was used for statistical data processing. Categorical variables were expressed as numbers and percentages, while continuous variables were shown as the median and interquartile range (IQR). The data do not follow a normal distribution, as determined by the Kolmogorov–Smirnov test. Fisher’s exact test was implemented for comparing categorical variables. Univariate analysis was used to investigate the relationship between different clinical characteristics and OHIP-14. The Mann–Whitney U test and the Kruskal–Wallis test were conducted to compare two or more independent samples, respectively. Person’s correlation and Spearman’s rank correlation tests assessed the degree of association between parameters of interest. Multiple linear regression analysis was performed to estimate the effects of statistically significant variables on the OHIP-14 score, the dependent variable. The level of significance for all statistical tests was set to 0.05.

## 3. Results

### 3.1. Participants’ Characteristics

A total of 80 pSS patients with a median age of 65.5 (26–81) years were included in the research. The majority of the study subjects were women (96.2%). Groups were similar in terms of age, education level, employment, marital status, alcohol intake, and physical activity (*p* > 0.05). The median disease duration was 8.0 (1–30) years (there was a statistically significant difference between the groups, *p* < 0.05). Socio-demographic and clinical pSS data are illustrated in Table 1 and Table 2, respectively.

### 3.2. Oral Findings

Table 3 shows the types of oral lesions and symptoms present in the case group. The most prevalent oral manifestation was exfoliative cheilitis (36.3%), followed by periodontal disease (30%), dental caries (20%), aphthae (16.3%), and atrophic glossitis (12.5%). About 32.5% of patients reported a burning sensation in the mouth, especially the tongue, and 30% complained of chewing and swallowing difficulties. A statistically significant difference between the groups was observed regarding all periodontal indexes measured (*p* < 0.001), as seen in Table 4.

### 3.3. Oral Health-Related Quality of Life

**Figure 1:**
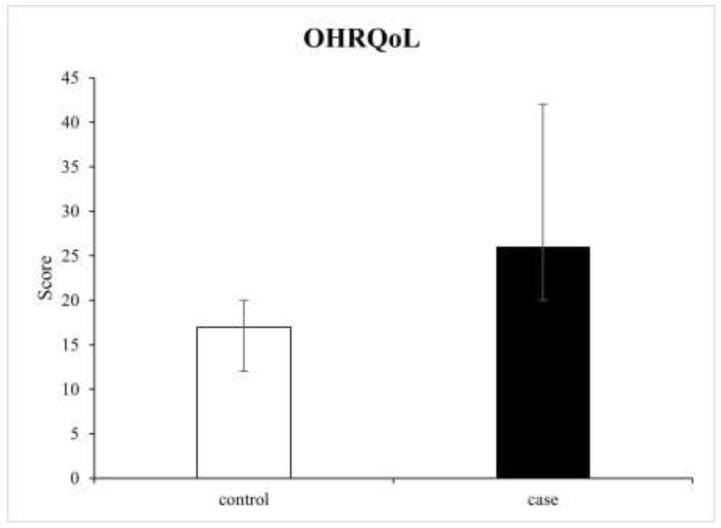
Comparison of the median and range values of the OHIP-14 scores between the groups.

**Figure 2:**
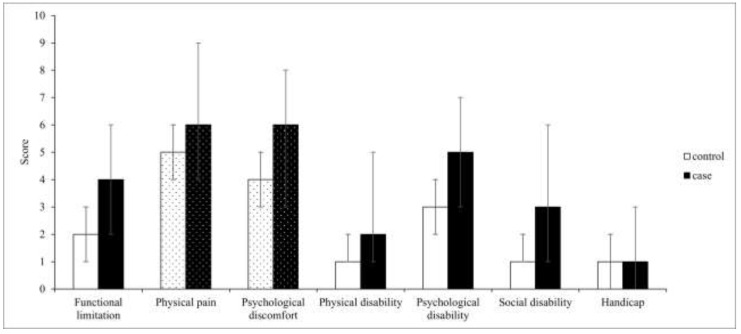
The median and range values of the OHIP-14 subscales’ scores (the dotted bars indicate subscales with the highest median values in both groups).

The OHIP-14 summary result was significantly higher in the case group than in the control group (26.0 (5.0) vs. 17.0 (4.0), respectively; *p* < 0.001), indicating that pSS patients with oral manifestations experienced greater impairment of the OHRQoL (Figure 1). A statistically significant difference was detected in all seven OHIP-14 subscales between the pSS patients with and without oral lesions (*p* < 0.001). Physical pain and psychological discomfort were domains with the highest median scores in both case and control groups (6.0 (1.0) and 6.0 (1.0) vs. 5.0 (1.0) and 4.0 (1.0), respectively). Outcomes for each dimension are depicted in Table 5 and Figure 2.

### 3.4. Factors Associated with OHRQoL

Patients with extraglandular symptoms tended to obtain significantly higher mean OHIP-14 results than those who did not have systemic involvement (24.0 (9.0) vs. 17.0 (3.0), respectively). This was also the case for the participants who received antimalarials and corticosteroids combined compared to chloroquine and hydroxychloroquine alone (25.0 (12.0) vs. 25.0 (9.0) vs. 17.0 (4.0), respectively). Table 6 shows the effects of different oral manifestations on the OHRQoL. The correlation between the periodontal parameters and the OHIP-14 score is shown in Table 7.

Multiple linear regression analysis was performed to assess the independent contribution of oral manifestations to the OHRQoL. Along with group allocation, all the cofactors that significantly correlated with OHIP-14 in the univariate analysis, such as systemic involvement, medication, and periodontal indexes, were put in the regression model. Due to collinearity between periodontal parameters, only CPITN was used, seeing that it provides the most comprehensive data on a patient’s periodontal status and treatment needs. Multiple linear regression analysis resulted in the model that explained 72.2% of the total variance (F = 47.783; *p* < 0.001) (Table 8).

## 4. Discussion

The main objective of our study was to evaluate the oral health status of pSS patients and determine its effect on different domains of OHRQoL. To date, there is limited information regarding this topic, so we aimed to reduce that knowledge gap by conducting the present research. Our results demonstrated that OHRQoL was substantially decreased in pSS subjects with oral lesions (for approximately 65% compared to participants without them), emphasizing their influence on all aspects of patients’ daily lives. Additionally, we found that oral manifestations were significantly and independently associated with OHIP-14 scores.

Oral health is an integrative part of general health and, thus, may profoundly impact the individual’s physical, psychological, and social functioning. OHRQoL represents a multidimensional concept created to measure how oral diseases affect patients’ well-being [16].

Saliva has a fundamental role in preserving oral homeostasis as it fulfills various important functions, including lubrication, pH buffering, antimicrobial activity, taste perception, and wound healing [17]. The prolonged decline in saliva quantity and quality during pSS may lead to the development of numerous lesions and symptoms in the oral cavity [6]. Some of the most frequent ones are candidiasis, traumatic lesions, aphthae, swallowing difficulties, and taste alteration, as reported by a recent systematic review [6]. Different research showed that patients with pSS had more commonly dysgeusia, burning sensation in the mouth, and halitosis compared to the controls, alongside impaired chemosensory and salivary functions [18]. Similarly, a comparative cross-sectional study comprising 58 pSS participants and 55 age- and gender-matched healthy controls demonstrated that the majority of pSS patients experienced chemosensory dysfunction, ageusia for basic tastes, halitosis, and burning sensations in the tongue [8]. Another research observed that pSS subjects had a high occurrence of lip dryness, exfoliative cheilitis, angular cheilitis, and small aphthae [19]. The findings of one cross-sectional study indicated that pSS patients often complained of swallowing and speaking difficulties, frequent fungal infections, and recurrently swollen salivary glands [10]. Our results revealed that our participants had similar signs and symptoms to those aforementioned. Exfoliative cheilitis was the most prevalent oral manifestation, followed by periodontal disease, dental caries, aphthae, and atrophic glossitis, while patients most commonly reported burning sensations in the tongue, as well as chewing and speaking problems. Regarding periodontal indexes, we observed significantly higher values in the patients presenting with oral manifestations. On the contrary, a study performed in southern China detected no difference between subjects with pSS and healthy controls, which might suggest better oral hygiene behavior of their pSS patients or more regular dental visits [20].

The OHIP-14 questionnaire, developed by Slade and Spencer in 1997, was used to assess the influence of oral alterations on patients’ well-being [21]. It is one of the most widely applied instruments for measuring the contribution of oral health to various aspects of people’s quality of life, such as physical, psychological, and social domains [22]. Our results showed that pSS subjects with oral lesions obtained significantly higher scores on the OHIP-14 index, indicating that their OHRQoL is worse by approximately 65% compared to patients without them. This discovery has important clinical implications as improving oral health status would possibly lead to remarkable OHRQoL enhancement and, consequently, greater life satisfaction. Other authors came to similar conclusions. One cross-sectional study demonstrated that oral distress is substantially increased in pSS, and that xerostomia-related symptoms pose a great burden to patients [10]. Another research study also reported a poor OHRQoL in 39 pSS subjects [5]. In a multicentric descriptive study, participants with pathological oral signs recorded significantly higher OHIP-14 results than those without them, which is in line with our findings [14]. Research conducted in Japan monitored changes in OHRQoL of pSS patients over a three-year period. It revealed a significant increase in their OHIP-14 scores compared to the initial assessment, especially in relation to the physical pain and psychological discomfort subscales [7]. Similarly, the findings of a recent study showed that OHRQoL in pSS seemed to be frequently impaired, with physical pain and psychological discomfort being the most affected OHIP-14 dimensions, which coincided with our results [23].

We analyzed variations in OHIP-14 scores based on the different characteristics of the study participants. Our results revealed that oral manifestations, extraglandular involvement, medication, and periodontal indexes were significantly associated with OHIP-14, which no other study has reported so far. Oral lesions are most likely the essential factor contributing to decreased OHRQoL in pSS patients. We demonstrated that patients who developed systemic complications during the course of the disease tended to have poorer OHRQoL. Our findings differ from the results of the only research that explored this relationship in which no correlation was found between extraglandular manifestations and OHIP-14 [14]. The type of medication used also affected participants’ OHRQoL. Our data indicated that subjects on hydroxychloroquine therapy recorded better OHIP-14 results in contrast to patients who were prescribed chloroquine or the combination of antimalarials and corticosteroids. To the best of our knowledge, no studies have investigated this topic up to now, so we consider this result a particular novelty of the present research. Our results also disclosed a significant association between periodontal indexes and OHIP-14 scores. They are important indicators of periodontal disease, which eventually leads to gingival recessions, tooth migration, and substantial tooth loss, all responsible for detrimental effects on all aspects of patients’ lives [20,24,25].

The main limitation of the present study is the small sample size due to a relatively low incidence of pSS in the general population and the ongoing COVID-19 pandemic making patients’ enrollment complicated. Further, the participants were recruited from only one rheumatology clinic registry, as it is the sole specialist unit for patients with connective tissue disorders in Kragujevac. Another limitation worth mentioning is that we used an OHRQoL measure of generic nature, which might not address all oral problems that pSS patients face daily. However, our plan is to conduct longitudinal research that would encompass a greater number of subjects with different clinical characteristics and whose quality of life would be evaluated by the disease-specific questionnaire, such as the PSS-QoL, which was recently validated in the Serbian language [26,27].

## 5. Conclusions

Participants with pSS accompanied by oral manifestations show significantly more reduced OHRQoL, especially concerning physical pain and psychological discomfort, compared with those who do not experience the same problems within the oral cavity. These findings emphasize the importance of oral diseases for patients’ well-being and have significant implications for routine clinical practice. Therefore, it is essential for dentists to be included in the multidisciplinary teams treating pSS, considering their pivotal position in identifying initial pSS signs early and referring a patient to a complete rheumatological assessment. Furthermore, regular dental appointments should be incorporated as a central part of the pSS management plan as they are required for timely diagnosis and successful treatment of different oral lesions and symptoms. That would improve not only the patients’ oral health, but also their OHRQoL. Future research in this area should explore the impact of different oral manifestations on the OHRQoL and investigate the efficacy of novel treatment options for oral lesions in pSS patients.

## Figures and Tables

**Table 1:** Socio-demographic characteristics of the patients.

Variables	Case Group (N = 40)	Control Group (N = 40)
Sex [*n* (%)] Males Females		
	2 (5.0) 38 (95.0)	1 (2.5) 39 (97.5)
Education [*n* (%)] Elementary school High school University degree Doctoral degree		
	9 (22.5) 18 (45.0) 11 (27.5) 2 (5.0)	9 (22.5) 20 (50.0) 10 (25.0) 1 (2.5)
Employment [*n* (%)] Employed Unemployed Retired		
	4 (10.0) 12 (30.0) 24 (60.0)	3 (7.5) 15 (37.5) 22 (55.0)
Marital status [*n* (%)] Single In a relationship/married Divorced Widowed		
	1 (2.5) 29 (72.5) 3 (7.5) 7 (17.5)	3 (7.5) 26 (65.0) 2 (5.0) 9 (22.5)
Alcohol [*n* (%)] Never Sometimes Regularly		
	36 (90.0) 4 (10.0) 0 (0.0)	39 (97.5) 1 (2.5) 0 (0.0)
Physical activity [*n* (%)] Never Sometimes Regularly		
	30 (75.0) 8 (20.0) 2 (5.0)	29 (72.5) 7 (17.5) 4 (10.0)
Disease duration [median (IQR)]	10 (13.0)	7 (8.0)

IQR—interquartile range.

**Table 2:** pSS-related characteristics of the patients.

Variables	Case Group (N = 40)	Control Group (N = 40)
pSS systemic manifestations [*n* (%)]		
Skin involvement	6 (15.0)	1 (2.5)
Musculoskeletal involvement	37 (92.5)	24 (60.0)
Renal involvement	3 (7.5)	2 (5.0)
Lung involvement	4 (10.0)	2 (5.0)
PNS involvement	9 (22.5)	3 (7.5)
Hematological involvement	3 (7.5)	3 (7.5)
Gastrointestinal involvement	2 (5.0)	0 (0.0)
Endocrine involvement	3 (7.5)	8 (20.0)
Immunological involvement	7 (17.5)	1 (2.5)
pSS serological findings [*n* (%)]		
RF+	29 (72.5)	25 (62.5)
ANA+	36 (90.0)	31 (77.5)
pSS medication [*n* (%)]		
Chloroquine	29 (72.5)	16 (40.0)
Hydroxychloroquine	1 (2.5)	18 (45.0)
Antimalarials + corticosteroids	10 (25.0)	6 (15.0)

pSS—Primary Sjögren Syndrome; PNS—peripheral nervous system; RF—rheumatoid factor; ANA—antinuclear antibodies.

**Table 3:** Frequency of oral lesions and symptoms in pSS patients.

Oral Lesions and Symptoms	Yes *n* (%)	No *n* (%)
Exfoliative cheilitis	29 (36.3)	51 (63.7)
Angular cheilitis	6 (7.5)	74 (92.5)
Aphthae	13 (16.3)	67 (83.7)
Traumatic lesions	8 (10.0)	72 (90.0)
Periodontal disease	24 (30.0)	56 (70.0)
Dental caries	16 (20.0)	64 (80.0)
Geographic tongue	3 (3.8)	77 (96.2)
Coated tongue	8 (10.0)	72 (90.0)
Atrophic glossitis	10 (12.5)	70 (87.5)
Denture stomatitis	9 (11.3)	71 (88.7)
Erythematous candidiasis	3 (3.8)	77 (96.2)
Lichen planus	4 (5.0)	76 (95.0)
Generalized stomatitis	5 (6.3)	75 (93.7)
Chewing difficulties	24 (30.0)	56 (70.0)
Swallowing difficulties	24 (30.0)	56 (70.0)
Speaking difficulties	10 (12.5)	70 (87.5)
Denture-wearing difficulties	15 (18.8)	65 (81.2)
Burning sensation	26 (32.5)	54 (67.5)
Taste alteration	20 (25.0)	60 (75.0)

**Table 4:** Comparison of periodontal indexes between the groups.

Periodontal Indexes ^a^	Case Group Median (IQR)	Control Group Median (IQR)	All Patients Median (IQR)
CPITN *	2.67 (0.67)	1.50 (0.83)	2.17 (1.30)
PI *	1.96 (0.56)	0.66 (0.38)	1.29 (1.30)
GI *	1.77 (0.59)	0.83 (0.21)	1.37 (0.96)
SBI *	2.02 (0.74)	0.41 (0.15)	0.56 (1.64)

CPITN—Community Periodontal Index of Treatment Need; PI—plaque index; GI—gingival index; SBI—sulcus bleeding index; IQR—interquartile range; * *p* < 0.001; ^a^ Mann–Whitney U test.

**Table 5:** OHIP-14 domains’ scores.

OHIP-14 Domains ^a^	Case Group Median (IQR)	Control Group Median (IQR)	All Patients Median (IQR)
Functional limitation *	4.0 (1.0)	2.0 (0.0)	3.0 (2.0)
Physical pain *	6.0 (1.0)	5.0 (1.0)	6.0 (1.0)
Psychological discomfort *	6.0 (1.0)	4.0 (0.0)	5.0 (2.0)
Physical disability *	2.0 (0.0)	1.0 (1.0)	1.0 (1.0)
Psychological disability *	5.0 (2.0)	3.0 (1.0)	4.0 (2.0)
Social disability *	3.0 (1.0)	1.0 (1.0)	2.0 (2.0)
Handicap *	1.0 (1.0)	1.0 (1.0)	1.0 (0.0)

OHIP-14—Oral Health Impact Profile-14; IQR—interquartile range * *p* < 0.001; ^a^ Mann–Whitney U test.

**Table 6:** Oral manifestations and OHIP-14.

Oral Manifestations ^a^	OHIP-14 Score Median (IQR)
Exfoliative cheilitis ** Yes No	26.0 (5.0) 18.0 (5.0)
Angular cheilitis * Yes No	26.0 (8.0) 20.0 (9.0)
Aphthae ** Yes No	27.0 (4.0) 19.0 (8.0)
Traumatic lesions * Yes No	26.0 (6.0) 19.5 (9.0)
Periodontal disease ** Yes No	26.0 (4.0) 18.0 (8.0)
Caries * Yes No	25.5 (3.0) 19.0 (9.0)
Coated tongue * Yes No	26.5 (5.0) 19.5 (9.0)
Atrophic glossitis ** Yes No	30.0 (5.0) 19.0 (9.0)
Denture stomatitis * Yes No	27.0 (5.0) 19.0 (9.0)
Generalized stomatitis * Yes No	29.0 (12.0) 20.0 (9.0)
Chewing difficulties ** Yes No	27.0 (5.0) 18.0 (8.0)
Swallowing difficulties ** Yes No	26.5 (5.0) 18.0 (7.0)
Speaking difficulties * Yes No	28.0 (6.0) 19.0 (9.0)
Denture-wearing difficulties ** Yes No	27.0 (6.0) 19.0 (9.0)
Burning sensation ** Yes No	26.5 (5.0) 18.0 (7.0)
Taste alteration ** Yes No	26.0 (5.0) 17.0 (4.0)

OHIP-14—Oral Health Impact Profile-14; IQR—interquartile range; * *p* < 0.05; ** *p* < 0.001; ^a^ Mann–Whitney U test.

**Table 7:** The correlation between the periodontal parameters and the OHIP-14.

Variable ^a^	CPITN *	PI *	GI *	SBI *
OHIP-14	0.600	0.763	0.720	0.681

OHIP-14—Oral Health Impact Profile-14; CPITN—Community Periodontal Index of Treatment Need; PI—plaque index; GI—gingival index; SBI—sulcus bleeding index; * *p* < 0.001; ^a^ Spearman’s rank correlation test.

**Table 8:** Multiple linear regression analysis of factors influencing the OHIP-14.

Variables ^a^	B	β	95% Confidence Interval
Group allocation *	9.112	0.731	6.924–11.301
Systemic involvement	1.236	0.066	−1.311–3.782
Medication	0.008	0.001	−0.971–0.987
CPITN	1.195	0.144	−0.176–2.566

OHIP-14—Oral Health Impact Profile-14; CPITN—Community Periodontal Index of Treatment Need; * *p* < 0.001; ^a^ Multiple linear regression analysis.

## Data Availability

The data presented in this study (including the Serbian and English versions of the OHIP-14 questionnaire) are available on request from the corresponding authors.
